# Acclimation temperature changes spermatozoa flagella length relative to head size in brown trout

**DOI:** 10.1242/bio.039461

**Published:** 2019-07-08

**Authors:** Miriam Fenkes, John L. Fitzpatrick, Holly A. Shiels, Robert L. Nudds

**Affiliations:** 1 University of Manchester, Faculty of Biology, Medicine and Health, Manchester M13 9PL, UK; 2 Department of Zoology/Ethology, Stockholm University, SE-10691 Stockholm, Sweden

**Keywords:** Climate change, Reproduction, Salmonid, Sperm quality, Sperm morphology, Temperature

## Abstract

Temperature is a ubiquitous environmental factor affecting physiological processes of ectotherms. Due to the effects of climate change on global air and water temperatures, predicting the impacts of changes in environmental thermal conditions on ecosystems is becoming increasingly important. This is especially crucial for migratory fish, such as the ecologically and economically vital salmonids, because their complex life histories make them particularly vulnerable. Here, we addressed the question whether temperature affects the morphology of brown trout, *Salmo trutta* L. spermatozoa. The fertilising ability of spermatozoa is commonly attributed to their morphological dimensions, thus implying direct impacts on the reproductive success of the male producing the cells. We show that absolute lengths of spermatozoa are not affected by temperature, but spermatozoa from warm acclimated *S. trutta* males have longer flagella relative to their head size compared to their cold acclimated counterparts. This did not directly affect sperm swimming speed, although spermatozoa from warm acclimated males may have experienced a hydrodynamic advantage at warmer temperatures, as suggested by our calculations of drag based on head size and sperm swimming speed. The results presented here highlight the importance of increasing our knowledge of the effects of temperature on all aspects of salmonid reproduction in order to secure their continued abundance.

## Abstract (precis)

**Summary:** Two different acclimation temperatures cause divergence in brown trout sperm morphometry through changes in the relative head surface area to flagellum length ratio.

## INTRODUCTION

As ectotherms, fish are directly influenced by the temperature of their environment. This makes water temperature one of the most ubiquitous of environmental factors affecting fish physiological processes, including development, growth, metabolic scope and reproduction (Brett, 1971; Casselman, 2002). Air temperatures directly affect the temperature of freshwater systems (IPCC, 2013; Isaak et al., 2010; Jonkers and Sharkey, 2016; van Vliet et al., 2011), making these habitats and their inhabitants particularly susceptible to the effects of global warming through human-induced, accelerated climate change (Almodóvar et al., 2012; IPCC, 2014). Migratory fish are especially affected by changes in water temperatures due to their complex life histories and the dependency of successive life stages on favourable thermal conditions (Crozier et al., 2008; Mathes et al., 2010). The predicted, continued increase in global air temperatures over the next century (IPCC, 2013) therefore gives cause for concern regarding the reproduction, and ultimately persistence, of migratory fish species, including the ecologically and socio-economically important salmonid family.

Salmonids perform anadromous or potamodromous reproductive migrations to their natal spawning grounds (Hinch et al., 2005). As capital breeders, they migrate in a catabolic state, relying entirely on endogenous energy reserves to fuel final maturation, migration and reproduction (Kinnison et al., 2003). This metabolic restriction can create direct energetic trade-offs between different facets of reproduction, especially when energy expenditure is altered by environmental factors such as temperature (reviewed by Fenkes et al., 2016). Therefore, salmonids represent ideal models for studying the impacts of temperature alterations on migratory fish reproduction.

Increased river water temperatures increase the metabolic costs of locomotion in fish, affecting the initiation (Cooke et al., 2008; Juanes et al., 2004; Quinn and Adams, 1996; Robards and Quinn, 2002), progress (Berman and Quinn, 1991; Goniea et al., 2006; High et al., 2006) and ultimately the success of salmonid migrations (i.e. pre-spawning mortality; Hinch et al., 2012). While these effects are well established, the sub-lethal consequences of thermal challenges for post-migratory reproductive behaviour and physiology remain poorly understood (Fenkes et al., 2016; Nadeau et al., 2010). Recently, it was shown that sperm swimming speed (a reliable predictor of fertilisation success; Simmons and Fitzpatrick, 2012) of brown trout, *Salmo trutta*, is reduced in males that are acclimated to increased water temperatures, compared to males kept at temperatures normally experienced during the reproductive season of the species (Fenkes et al., 2017). The authors (Fenkes et al., 2017) attributed their results to a potential temperature-mediated delay in maturation and sperm production, but found that warm acclimated males compensated for this delay later on in the spawning season, when temperature-dependent differences in sperm swimming speed were no longer observed.

Differences in sperm swimming speed are commonly attributed to differences in sperm morphology, as sperm size can be positively correlated with swimming speed (Fitzpatrick et al., 2010; Simmons and Fitzpatrick, 2012). However, the relationship between sperm form and function is far from clear (Humphries et al., 2008): while increased flagellum length does theoretically increase thrust production, sperm swimming speed is negatively impacted by the drag force acting on the sperm head. It may therefore be the relative sizes of sperm cells' constituent parts, rather than their absolute lengths, which can reliably predict sperm swimming speeds (Humphries et al., 2008). Sperm morphology has been shown to vary throughout the reproductive season in several fish species [e.g. *Barbus barbus* (Alavi et al., 2008); *Scophthalmus maximus* (Suquet et al., 1998)] and these changes may be attributed to ageing and maturation of the sperm (Alavi et al., 2008). In light of the known effects of acclimation temperature on maturation, the question arises whether temperature directly affects sperm morphology in fish such as salmonids.

Spermatogenesis and sperm release occur in distinct cycles in salmonid fishes. Early spermatozoa can be released before peak maturation, and residual spermatozoa remain afterwards, before being resorbed within the testicular lobe, beginning another cycle of spermatogenesis and release (e.g. *Oncorhynchus mykiss*; Billard, 1986). Morphological differences may exist between early, ripe and late spermatozoa (Alavi et al., 2008; Suquet et al., 1998). Given that exposure to increased acclimation temperature during the sperm maturation process can delay peak maturation (Fenkes et al., 2017; Lahnsteiner and Leitner, 2013) and may deplete stored energy, morphological differences in spermatozoa might therefore be expected in semen samples from differentially acclimated fish. Increased temperatures have been shown to reduce the size and speed of spermatozoa in a tropical fish (*Poecilia reticulata*; Breckels and Neff, 2013). However, the effects of temperature on the morphology of salmonid sperm have not been investigated to date.

Here, the morphology of spermatozoa from cold and warm acclimated male *S. trutta* was compared. This study described and identified different types of morphological deformations in trout sperm, and compared their prevalence in semen samples from differentially acclimated donor males. Sperm head length (*L*_H_, µm), head width (*W*_H_, µm), flagellum length (*L*_F_, µm), total sperm length (*L*_T_, µm) and head surface area (*A*_H_, µm^2^) of photographed sperm cells were measured. Flagellum length to head length ratio (*L*_F_/*L*_H_, dimensionless) and flagellum length to head surface area ratio (*L*_F_/*A*_H_, µm^−1^) were additionally calculated to be used as morphological predictors of sperm swimming speed. In accordance with previous findings (Breckels and Neff, 2013), a difference in absolute measurements of sperm constituent parts was predicted to exist between the acclimation groups, with a possible reduction in flagellum and total sperm lengths for warm acclimated males. Based on previous evidence (Humphries et al., 2008), a positive correlation between flagellum length to head length and/or head surface area ratio and sperm swimming speed was hypothesised. The temperature of water determines its viscosity (Korson et al., 1969), which in turn has significant impacts on the drag forces experienced by microscopic, moving objects such as sperm cells, because they operate at low Reynolds numbers (Taylor, 1951). Here, drag force (*D*, N) experienced by sperm heads at different water temperatures was calculated theoretically using Stoke's law and compared between sperm cells from warm and cold acclimated male donors. It was hypothesised that drag would decrease when the spermatozoa were swimming in higher water temperature; however, due to a lack of previous evidence, we could not reliably predict whether the acclimation temperature of the male trout would influence drag.

## RESULTS

### Deformed spermatozoa

**Fig. 1.**
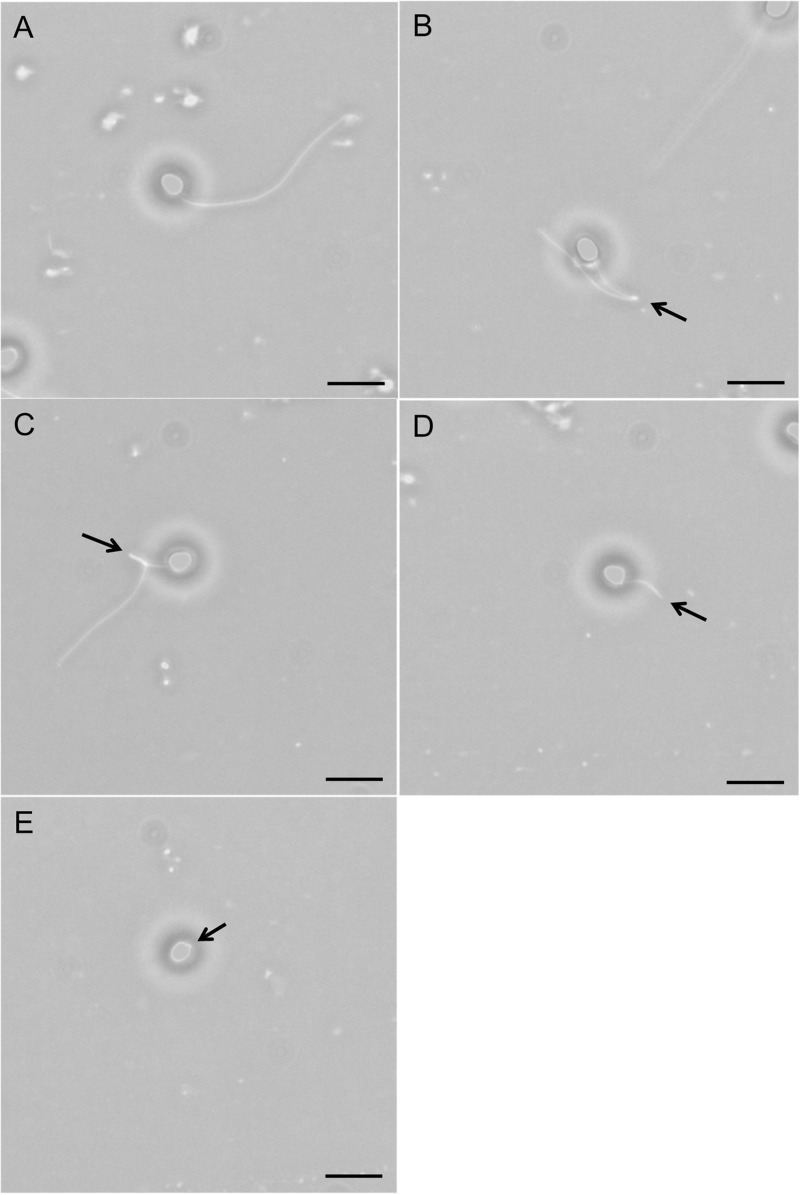
**Deformities of *S. trutta* sperm cells.** (A) Normal cell; (B) ‘kink’ cell; (C) ‘coil’ cell; (D) ‘short’ cell and (E) ‘tailless’ cell. Photographs were taken at 400× magnification (phase contrast microscopy) for illustrative purposes; counts were conducted using 400× magnification dark field microscopy. Scale bars: 0.01 mm.

Four types of morphological deformation were identified and spermatozoa were categorised accordingly either as ‘normal’ (no deformation; Fig. 1A), ‘kink’ (Fig. 1B), ‘coil’ (Fig. 1C), ‘short’ (Fig. 1D) or ‘tailless’ (Fig. 1E). Kink cells were characterised by a bend in the flagellum. Coil cells showed signs of repeated flagellum bending resulting in knotting. Tailless cells lacked a flagellum altogether and short cells had drastically shortened flagella compared to a normal cell. The proportion of different types of morphologically deformed sperm cells did not differ between cold and warm acclimated samples (Table 1).

**Table 1.**
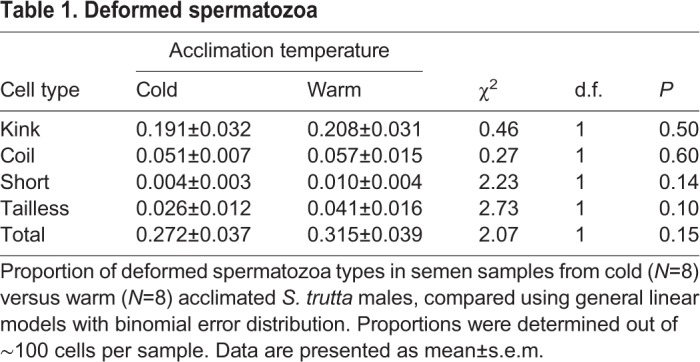
**Deformed spermatozoa**

### Sperm morphology

**Fig. 2.**
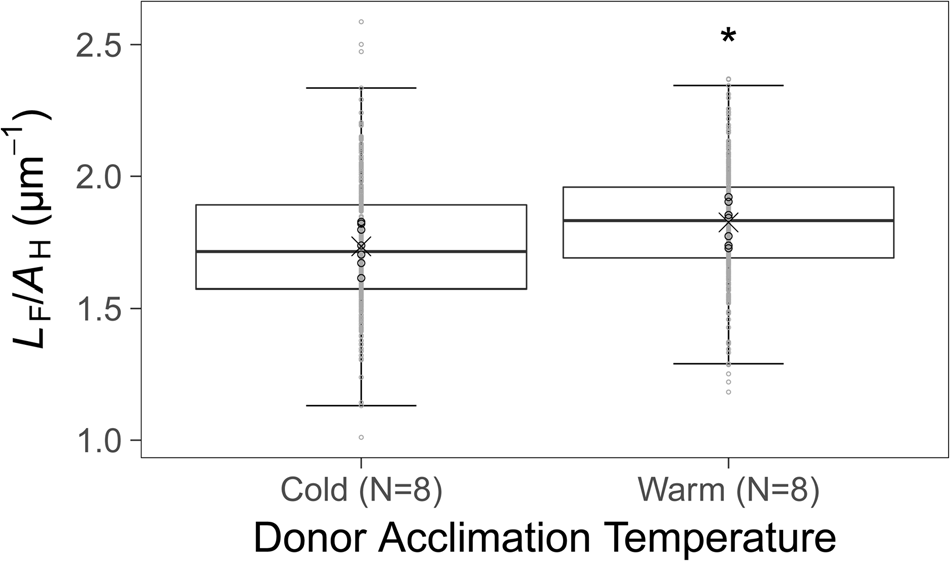
**Acclimation temperature effect on sperm flagellum length to head surface area ratio, *L*_F_/*A*_H_.** *L*_F_/*A*_H_ ratio (µm^−1^) of 40 sperm cells measured per cold (8°C, *N*=8) versus warm (13°C, *N*=8) acclimated *S. trutta* male semen sample. Data are presented as median (−) mean (X), IQR (box) and 95% CI (whiskers). Grey circles show raw data and black circles represent individual means for each male. The asterisk denotes significant (*P*<0.05) difference, determined by a general linear mixed effects model.

Sperm head surface area (*A*_H_, µm^2^), flagellum length (*L*_F_, µm), total length (*L*_T_, µm) and flagellum length to head length ratio (*L*_F_/*L*_H_) did not differ between cold and warm acclimated male sperm cells (Table 2). However, a significant increase in sperm flagellum length to head surface area ratio (*L*_F_/*A*_H_, µm^−1^) was evident for warm acclimated male sperm cells compared to cold acclimated male sperm cells (Table 2, Fig. 2).

**Table 2.**
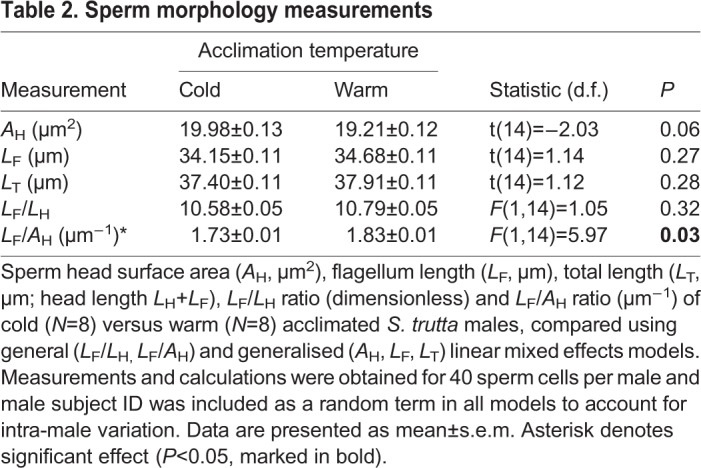
**Sperm morphology measurements**

### Sperm morphology effects on sperm swimming speed

Sperm flagellum length to head length ratio (L_F_/*L*_H_) did not affect sperm swimming speed at 10 s post activation in either sperm activation temperature (8°C or 13°C). *L*_F_/*A*_H_ ratio and acclimation temperature also did not impact on sperm swimming speed at either activation temperature, and no interaction between the terms was detected (Table 3).

**Table 3.**
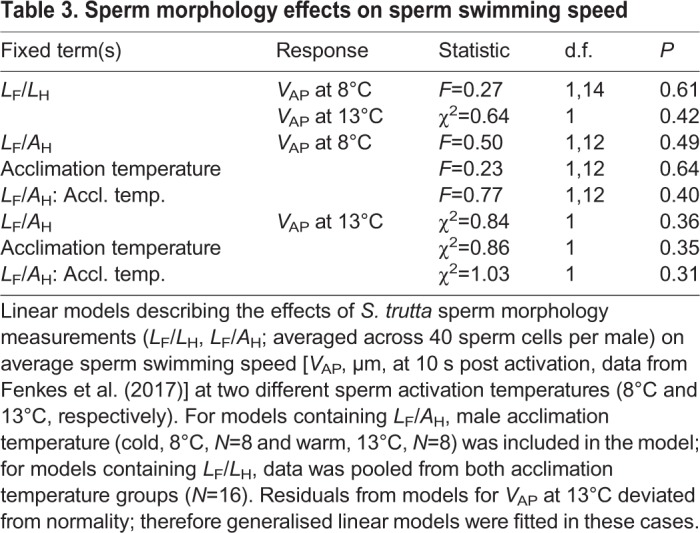
**Sperm morphology effects on sperm swimming speed**

### Drag force

Drag force, *D*, on sperm heads was not affected by the acclimation temperature of the donor males (Table 4). Drag force decreased with increasing sperm activation temperature (Table 4) and this effect was similar in both male acclimation temperature groups, as evidenced by the non-significant interaction between these terms (Table 4). However, *post-hoc* least squares means analysis revealed that theoretical drag was significantly decreased at 13°C compared to 8°C activation temperature in warm acclimated males (t=2.47; d.f.=14; *P*=0.03), but not in cold acclimated males (t=1.66; d.f.=14; *P*=0.12).

**Table 4.**
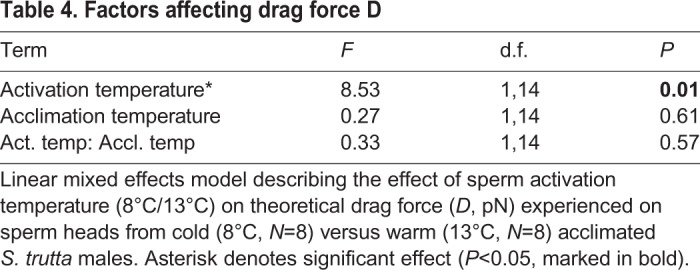
**Factors affecting drag force D**

## DISCUSSION

While no differences in absolute size of sperm morphological parameters were detected, sperm from warm acclimated males had significantly higher sperm flagellum length to head surface area ratios than their cold acclimated counterparts. Morphological parameters did not affect sperm swimming speed, but theoretical drag (driven by smaller head size) experienced on the sperm heads was decreased at higher temperature (when the viscosity of water is lower), for sperm from warm acclimated males. Acclimation temperature did not affect the frequency of flagellar deformities in the *S. trutta* sperm.

Exposure to increased temperatures has previously been shown to result in an increase of sperm cells with pyriform (short, narrow, posteriorly compressed) heads (Merino sheep, *Ovis aries*, Rathore, 1968; Duroc boars, *Sus scrofa*, Suriyasomboon et al., 2005; Holstein bulls, *Bos taurus*, Vogler et al., 1993), as well as an increase in the prevalence of tailless spermatozoa (Rathore, 1968; Vogler et al., 1993) and acrosomal abnormalities (Rathore, 1968). Other flagellar abnormalities such as coiling and bending have previously been identified, but reported changes in their frequency in response to temperature are contradictory. Suriyasomboon et al. (2005) reported no change in the number of Duroc boar spermatozoa with flagellar abnormalities, while an increase with temperature was reported in Holstein bulls (Vogler et al., 1993). However, our current understanding of the effects of temperature on sperm morphology, especially in externally fertilising ectotherms, is limited. We did not identify head abnormalities in *S. trutta* spermatozoa, but the semen samples contained high numbers (approximately 30%) of spermatozoa with flagellar abnormalities as well as altogether tailless cells. While it is possible that these abnormalities, especially partial or complete loss of the flagellum, may be preservation artefacts, 30% is similar to previous measurements of percentages of non-motile cells in freshly extracted *S. trutta* semen (Fenkes et al., 2017).

In one of the first accounts of a temperature effect on sperm morphology in an ectotherm, *Drosophila melanogaster* spermatozoa were found to be longer (increased total length) for males kept at higher temperatures (Blanckenhorn and Hellriegel, 2002). The only previous study investigating the effect of temperature on sperm morphology in a fish showed that increased acclimation temperature was associated with a reduction in sperm flagellum length and swimming speed (Trinidadian guppy, *P. reticulata*, Breckels and Neff, 2013). Neither study (Blanckenhorn and Hellriegel, 2002; Breckels and Neff, 2013) investigated sperm head and flagellum morphology separately. Tropical fish species, such as *P. reticulata* generally operate at a much narrower thermal range compared to temperate species (Breckels and Neff, 2013) such as the brown trout used in our study. The discrepancy in this study's results and the findings for *P. reticulata* sperm may indicate that temperate species are more resistant to the effects of warming on their spermatozoa morphology. Therefore, longer-term exposure than implemented in this present study, perhaps throughout development, may be necessary to induce radical changes in trout spermatozoa morphology and motility comparable to those observed in tropical fish.

Flagellum length to head surface area ratio is theoretically a reliable predictor of sperm swimming speed (Humphries et al., 2008). However, the findings of this study did not demonstrate a difference in the average speed of spermatozoa from samples with different average flagellum length to head length or flagellum length to head surface area ratios for either acclimation temperature treatment. Sperm flagellum length and total length have previously been linked to decreased sperm longevity [e.g. *Gadus morhua* (Tuset et al., 2008); *Salmo salar* (Gage et al., 2002)], but, similar to the present study, no effect on initial sperm swimming speed was detected. In *S. salar*, mid-piece size and ATP content were positively correlated, as were sperm flagellum length and sperm energy charge (Vladić et al., 2002). Thus, a longer flagellum appears to require higher, more effective ATP provision in order to allow the sperm to reach an egg, and this is provided by a larger mid-piece, containing more active mitochondria (Vladić et al., 2002). Here, however, warm acclimated *S. trutta* males had relatively longer flagella and smaller heads (containing the mid-piece) than cold acclimated males, as evident in the increased *L*_F_/*A*_H_ ratio.

Within the low Reynolds number environment in which sperm operate, viscosity is the dominating force determining speed, while inertia is negligible. Decreased head size or a change in shape could provide an advantage as drag force on the head is congruently reduced. The results of this study show that the theoretical drag experienced on the sperm head was lower at higher activation temperatures for warm acclimated but not cold acclimated males. All terms except head radius and speed are constant in Stoke's law (Eqn 3). Therefore, if *D* is reduced for warm acclimated males that have smaller heads (*a*), they must be swimming at a similar speed (*U*) to cold acclimated males. This suggests that warm acclimated males lack the power to generate the thrust required to take advantage of the reduced head drag. Therefore, the results support the idea that the warm acclimated males have a smaller power unit (mid-piece) as well as a smaller head. However, the changes in morphology (increased *L*_f_/*A*_h_ ratio) appear to allow the spermatozoa of warm acclimated males to increase their swimming speed to the level achieved by cold acclimated male sperm. What drives the need for the morphological change is not clear, but the energy constraints associated with higher acclimation temperature may not have allowed for the production of spermatozoa morphologically similar to those of cold acclimated males. Further in-depth investigations into the effects of short- and long-term exposure to increased temperature on salmonid spermatozoa morphology as well as cytophysiology (e.g. ATP content) throughout the reproductive season are needed to confirm these effects.

An additional explanation for our findings, and a caveat of this study, is that the effects of intra-male variation in sperm characteristics could have masked a possible link between spermatozoa morphology and swimming speed. As highlighted by Fitzpatrick et al. (2010), the typically high levels of variability in spermatozoa morphology and motility parameters within individual samples can mask length-velocity relationships at the intraspecific level. Therefore, while a positive relationship between sperm head size to flagellum length ratio has been described in other externally fertilising species (Simpson et al., 2014), and may also exist in trout, any link is likely to have been weakened because variation within ejaculates was not accounted for (Simpson et al., 2014).

### Conclusion

This study identified a change in the relative dimensions of salmonid spermatozoa in response to acclimation temperature. This change did not affect sperm motility, but had possible hydrodynamic consequences by affecting theoretical drag experienced by the moving cell. Currently, we do not know whether these findings are applicable to other teleost fish. Nevertheless, this study provides a foundation for future studies, and highlights the need to increase our limited understanding of the impacts of temperature across all aspects of migratory fish reproduction. Increasing knowledge of temperature driven trade-offs within and between each reproductive stage is essential if we are to maintain the abundance of migratory fish during the predicted changes to the global climate.

## MATERIALS AND METHODS

### Experimental setup

In October 2015, 3-year-old male *S. trutta* were obtained from Dunsop Bridge Trout Farm Ltd. (Clitheroe, UK) and individually PIT tagged (Biomark, Inc., Boise, ID, USA) upon arrival. A previous study utilised the same individuals, and detailed housing conditions are described therein (Fenkes et al., 2017). Briefly, individuals were housed at equal numbers in two outdoor tanks, under natural photoperiod and gradually (on average 0.4°C day^−1^ over 17 and 22 days, respectively) declining water temperature to induce maturation. One tank was then maintained at a ‘warm’ experimental temperature of 13°C, and the other at a ‘cold’ experimental temperature of 8°C. Until they ceased feeding at the onset of the spawning season, the trout were offered commercial trout pellets (Skretting, Trouw Ltd., Northwich, UK)] daily. Semen sampling for sperm swimming speed (data from Fenkes et al., 2017) and sperm morphological assessment (present study) was carried out after 4 weeks of differential temperature acclimation (8th and 9th December).

### Semen sampling

The semen samples used in the present study were collected and utilised in a previous study (Fenkes et al., 2017) and the sample collection protocol is detailed there. Briefly, males were lightly sedated via immersion in a buffered tricaine-methanesulfonate (MS-222) solution. Anaesthetised males were removed from the anaesthetic bath, the urogenital/anal region was dried, the bladder and bowel were emptied, and semen was carefully expressed by applying gentle pressure to both sides of the ventral mid line. Semen was captured directly into clean Eppendorf tubes; uncontaminated semen samples were immediately sealed and placed into an ice-cooled container. Samples contaminated with water, urine or faeces were discarded. Fish were moved into an oxygenated recovery bath before being transferred back into their holding tanks upon full recovery. Semen samples from *N*=8 cold and *N*=9 warm acclimated males (after 4 weeks of differential temperature acclimation) were used for sperm motility assessments in a previous study (Fenkes et al., 2017). For *N*=8 cold and *N*=8 warm acclimated males, a subsample was preserved in 10% neutral buffered formalin at a dilution of 1 part semen to 1000 parts formalin for the subsequent sperm morphology measurements described here. For this current study, sperm swimming speed data (from Fenkes et al., 2017) were used only from those males where associated sperm morphology measurements could be obtained.

### Ethical note

Experimental procedures were covered by a UK Home Office project licence (licence number 40/3584, licence holder H.A.S.) and were approved by the University of Manchester's ethical committee.

### Deformed spermatozoa identification and count

Each formalin-preserved semen sample was grid-scanned and spermatozoa were viewed at 400× magnification using dark field microscopy (UB 200i Microscope series, Proiser – Projectes i Serveis R+D S.L., Paterna, ES), scaled to a stage micrometre, using XIMEA CamTool Version 4.13 (XIMEA GmbH, Münster, D) and ImageJ 1.49v (http://imagej.nih.gov/ij) software. The first ∼100 cells in view were counted (if the number of cells in the last counted field of view caused the total to exceed 100, all cells in that field of view were counted, increasing the total number of cells counted accordingly) and different types of morphological deformation were identified and categorised. Aggregations of spermatozoa where individual cells were indiscernible were excluded from the count.

### Sperm morphology measurements

Sperm morphology measurements were subsequently obtained from sperm cells photographed at 400x magnification, as above. Cells to be photographed were chosen sequentially through a grid-scan of each mounted sample to avoid repeat recording. Deformed cells were excluded from measurements. Measurements of sperm head length (*L*_H_, µm; measured from flagellum insertion to head apex), sperm head width (*W*_H_, µm; measured centre-perpendicular to *L*_H_) and flagellum length (*L*_F_, µm) were obtained for 40 cells per male. The mid-piece was not measured, as it was too small to distinguish from the head using light microscopy, as is the case in most fishes (Gage et al., 2002). Using the above measurements, sperm total length (*L*_T_, µm; *L*_H_+*L*_F_) as well as head surface area (*A*_H_, µm^2^) were calculated. Under the assumption that the sperm head approximates a prolate spheroid (where the polar radius>equatorial radius), head surface area is given as:(1)
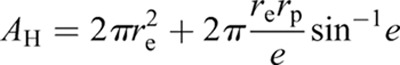
where *r*_e_ is the equatorial radius of the spheroid (0.5×head width *W*_H_), *r*_p_ is the polar radius of the spheroid (0.5×head length *L*_H_) and *e* is the ellipticity of the spheroid, given by:(2)
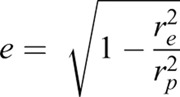
(adapted from Humphries et al., 2008). As suggested by Humphries et al. (2008), flagellum length to head length ratio (*L*_F_/*L*_H_, dimensionless) and flagellum length to head surface area ratio (*L*_F_/*A*_H_, µm^−1^) were calculated to be used as morphological predictors of sperm swimming speed.

### Sperm swimming speed assessment

As detailed in Fenkes et al. (2017), sperm swimming speed was measured as average path velocity (*V*_AP_, µm s^−1^) from video recordings of activated sperm obtained under 250× magnification, using an automated computer assisted sperm quality analysis plugin [CASA_automated plugin, www.ucs.mun.ca/~cfpurchase/CASA_automated-files.zip; see Purchase and Earle (2012) for further documentation] for ImageJ 1.49v (32-bit) (http://imagej.nih.gov/ij). Sperm from each male were activated with distilled water at both acclimation temperatures (‘activation temperature’; 8°C and 13°C). Sperm swimming speed has been shown to be a reliable predictor of fertilisation capacity (e.g. curvilinear velocity in *S. salar*; Gage et al., 2004). Here, sperm swimming speed (*V*_AP_) in each sample was recorded as an average of all sperm cells in the field of view every 2 s from 10 s after activation. Sperm swimming speeds recorded at 10 s after activation in both sperm activation temperature treatments (8°C and 13°C) of each sample (from cold and warm acclimated males) are used in this present study to assess the effects of warm acclimation and associated sperm morphology on sperm swimming speed.

### Drag

In addition to the above measurements and derived variables, theoretical drag force [*D*, Newton (N); Eqn 3] of sperm heads was compared between acclimation temperature groups. Drag force was calculated according to Stoke's Law (Dusenbery, 2009) as:(3)
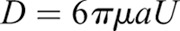
where *µ* is the dynamic viscosity (Pa s) of the water with which sperm were activated, *a* is the radius of the head (here, head width 0.5×*W*_H_, m) and *U* is the flow velocity relative to the head (here, *V*_AP_, m s^−1^). Literature values for dynamic viscosity were obtained from Korson et al. (1969): for our ‘cold’ activation water temperature (8°C), *µ* is the average value measured by Korson et al. (1969) for water at 5°C and 10°C, while for our ‘warm’ temperature (13°C), we used the average *µ* measured for water at 10°C and 15°C. For the flow velocity *U*, *V*_AP_ at 10 s post activation, measured at the respective activation temperatures (8°C or 13°C) by Fenkes et al. (2017) was used. As a result, theoretical values of drag on 40 sperm heads for each male were obtained at both activation water temperatures (i.e. 80 measurements in total per male).

Values were averaged across all 40 cells per male in each activation water temperature treatment and converted to pico Newtons (pN) for use in further analyses.

### Statistical analyses

All statistical analyses were conducted using R 3.3.1 GUI 1.68 Mavericks build (http://www.R-project.org/). Statistical significance levels are *P*<0.05.

### Deformed spermatozoa

To assess whether deformed spermatozoa counts differed between acclimation temperature treatments, generalised linear models with binomial error distribution were performed using deformed spermatozoa count/total cell count for each sample as response and donor acclimation temperature (cold/warm) as independent variable (car package 2.1-3; Fox and Weisberg, 2011).

### Sperm morphology

Mixed effects models were created to determine whether sperm morphology measurements differed between acclimation temperatures. Intra-male variation was taken into account by inclusion as a random effect in the models. Visual evaluation (quantile-quantile plots) of the residual distribution in the models for *L*_F_/*L*_H_ ratio and *L*_F_/*A*_H_ ratio gave no indication of significant deviations from normality and linear mixed effects models [lme4 package 1.1-12 (Bates et al., 2015) and lmerTest package 2.0-32 (https://CRAN.R-project.org/package=lmerTest)] were therefore fitted in these cases. However, quantile-quantile plots showed that the residuals in the models for the remaining measurements (head surface area *A*_H_, flagellum length *L*_F_ and total length *L*_T_) were not within confidence limits for normality. Generalised linear mixed effects models with penalised quasi-likelihood and log-normal error family [nlme package 3.1-128 (http://CRAN.R-project.org/package=nlme) and MASS package (Venables and Ripley, 2002)] were fitted in these cases.

### Sperm morphology effects on swimming speed

To test whether sperm morphology could predict sperm swimming speed, linear models (car package 2.1-3; Fox and Weisberg, 2011) were performed with average path velocity at 10 s post activation (*V*_AP_, µm s^−1^; data from Fenkes et al., 2017) as continuous response variable, and *L*_F_/*L*_H_ or *L*_F_/*A*_H_ (averaged across 40 spermatozoa per male, because only averages of speed were measured) as independent variables. Because *L*_F_/*A*_H_ differed between acclimation temperature groups (described above), acclimation temperature (cold/warm) was included as an additional independent variable in the respective models. Separate models were created for *V*_AP_ measured at 8°C and 13°C, respectively. Visual evaluation (quantile-quantile plots) of the residual distribution in the models for *V*_AP_ at 8°C gave no indication of significant deviations from normality and general linear models were fitted in those cases. However, quantile-quantile plots showed that the residuals in the models for *V*_AP_ at 13°C were not within confidence limits for normality and generalised linear models were fitted in those cases.

### Drag

To test whether drag on the sperm head (*D*, pN) differed between acclimation temperatures, a linear mixed effect model [lme4 package 1.1-12 (Bates et al., 2015) and lmerTest package 2.0-32 (https://CRAN.R-project.org/package=lmerTest)] was performed with drag force as the continuous response variable and male acclimation temperature (cold, 8°C and warm, 13°C) as well as activation temperature for sperm (cold, 8°C and warm, 13°C) as categorical independent variables. Male subject ID nested within acclimation temperature was included as a random effect term. Visual evaluation (quantile-quantile plots) of the residual distribution in the model gave no indication of significant deviations from normality. Least squares means [lmerTest package 2.0-32 (https://CRAN.R-project.org/package=lmerTest)] were calculated as *post-hoc* comparisons between factor levels.
